# Right Biceps Pseudo-Tumor from COVID-19 Vaccination

**DOI:** 10.3390/vaccines12020160

**Published:** 2024-02-03

**Authors:** Anas M. Abbas, Martha L. Caicedo, Timothy A. Damron

**Affiliations:** 1 Department of Orthopedic Surgery, SUNY Upstate Medical University, Syracuse, NY 13057, USA; 2 Department of Pathology and Laboratory Medicine, SUNY Upstate Medical University, Syracuse, NY 13210, USA

**Keywords:** biceps, COVID-19, hypersensitivity reaction, immunology, infectious disease, Moderna, oncology, orthopedic surgery, tumor, vaccine

## Abstract

Delayed hypersensitivity reactions (DHRs) have been reported in association with COVID-19 vaccines, particularly those that are mRNA-based. Classic DHRs result in induration, erythema, tenderness, and urticaria. However, soft tissue mass is an uncommon complication of a COVID-19 vaccination-associated DHR and is rarely reported in the literature. We present a case of a 49-year-old male who recognized a mildly painful, firm soft tissue mass within the biceps mimicking neoplasm six months after receiving the booster dose of the Moderna vaccine. Non-operative conservative treatment modalities, including heating pads, ice packs, acetaminophen, and ibuprofen, failed to improve the patient’s mass. The mass, which proved histologically to be an inflammatory pseudo-tumor, did not recur after complete excision. While there have been many reported cases of DHRs following COVID-19 vaccinations, we present this case to raise awareness of the development of pseudo-tumors as a possible, yet rare, clinical manifestation of DHRs following vaccination.

## 1. Introduction

In the United States, 97% of the coronavirus disease 2019 (COVID-19) vaccine doses that were administered were messenger RNA (mRNA)-based vaccines [1]. Initial fears of side-effects of the newly introduced mRNA vaccines contributed to vaccine hesitancy. As with any vaccine, COVID-19 vaccines have been associated with rare reports of immediate and delayed hypersensitivity reactions [2]. The incidence of COVID vaccine immediate hypersensitivity reactions (estimated to be 2.5–11.1 cases/million doses) is higher than the reports for conventional pediatric or adolescent vaccines (1.4 cases/million doses) [2]. Delayed hypersensitivity reactions (DHRs) have also been associated with COVID-19 vaccines, especially with the mRNA-based vaccines (mRNA-1273 (Moderna) > BNT162b2 (Pfizer)) [3]. The incidence of DHRs after receiving both doses of either Pfizer or Moderna vaccines is estimated to be 4.3% [4].

Delayed hypersensitivity reactions are adverse events defined as cutaneous complications that present after four hours post-vaccination [5]. The incidence of cutaneous reactions after receiving the first dose of the Moderna vaccine is 2.1%, with 17% of those affected also developing recurrent reactions after receiving the second dose [4]. Furthermore, 2.6% of patients reported adverse cutaneous reactions after receiving the second dose without developing adverse events from the first dose [4]. Symptoms of DHR from COVID-19 mRNA-based vaccines may present with various cutaneous reactions, such as morbilliform rash and bullous pemphigoid, with a median onset of eight days [5]. The classical morphology ranges from erythematous patches to large plaques, and lesion diameters typically range from 5 to 20 centimeters (cm) [5]. Symptoms commonly resolve after five days following nonoperative conservative treatment modalities such as corticosteroids or antihistamines [5].

As the number of vaccines administered continues to increase, so do reports of hypersensitivity reactions, which have been attributed to vaccine ingredients and injection techniques [2,6]. To our knowledge, there have only been two prior reports of granulomatous soft tissue mass (pseudo-tumor) reactions following COVID-19 vaccination [7,8]. We present a case of a patient who recognized a mass in the proximal-medial short head of the right biceps six months after receiving a booster dose of the Moderna vaccine. After failing local measures to decrease the swelling’s size, a complete excision was elected to remove the mass. We hope to raise awareness of the appearance of pseudo-tumors by taking into context a history of COVID-19 vaccination.

## 2. Case Presentation

A 49-year-old male presented to his primary care physician with a four-day history (6 months after receiving the booster dose) of a firm mass in the proximal-medial short-head of the right biceps muscle. He had no history of trauma prior to the recognition of the mass other than the injection of his Moderna vaccine booster dose administered into his right upper arm. Past medical history revealed no major medical, oncologic, rheumatologic, or orthopedic conditions. He had no known drug allergies. He described two lipomas in the right forearm that had remained unchanged for over 10 years. After each of his two initial Moderna vaccination doses, he experienced transient injection site soreness but no other acute or chronic symptoms following completion of the primary series.

Eight months after completing the primary series, the patient went to a walk-in vaccination center to receive a booster dose of the Moderna vaccine. Discussion with the patient revealed that the needle was inserted below the bulk of the deltoid muscle, approximately one finger breadth above the deltoid tuberosity. Immediately after receiving the booster dose, the patient developed constitutional symptoms, including diaphragmatic breathing, fatigue, and reduced exercise tolerance. After one week, the patient consulted his primary care physician, who diagnosed him with viral syndrome and recommended ibuprofen, fluids, and rest. However, these symptoms persisted over the next several months despite following the treatment protocol.

**Figure 1:**
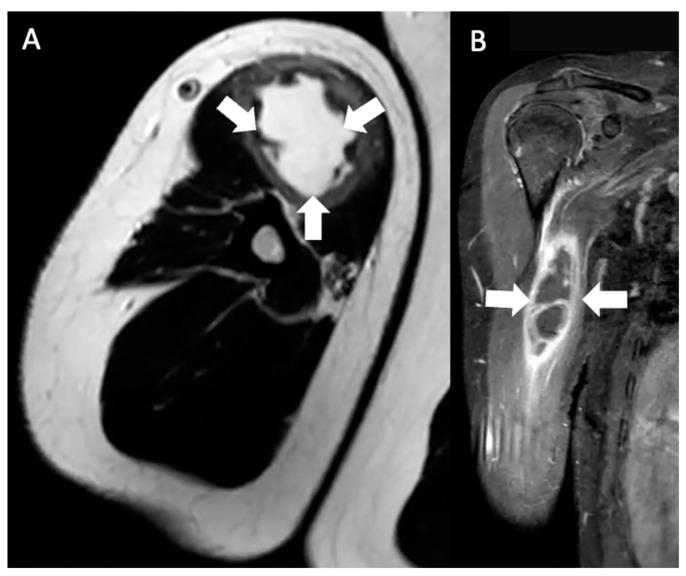
Multiplanar MRI of the right upper arm taken five days after identifying the mass and six months after receiving the booster dose. Axial T2 (**A**) and coronal T1W FS (**B**) images show an ill-defined fluid signal within the short head, right biceps muscle belly. The area involved extended from the muscle belly to the proximal humeral cortex, measuring approximately 7.4 × 3.7 × 2.6 cm (arrows).

Six months after the booster, the patient discovered a mass in the anteromedial right upper arm. He reported 3-out-of-10 pain in his right arm, but his main concerns were limited range of motion (ROM) attributed to a feeling of “heaviness” and his concern of a possible malignancy. The lump was described as a solid, “golf-ball-sized” mass without induration, warmth, discoloration, or urticaria. Four days after noticing the mass, he consulted his primary care physician, who ordered a magnetic resonance imaging (MRI) of his right arm that showed a 7.4 × 3.7 × 2.6 cm mass in the short head of the right biceps with mild hemorrhagic fluid abutting the humerus but with no evidence of cortical erosion or bone marrow signal change (Figure 1).

**Figure 2:**
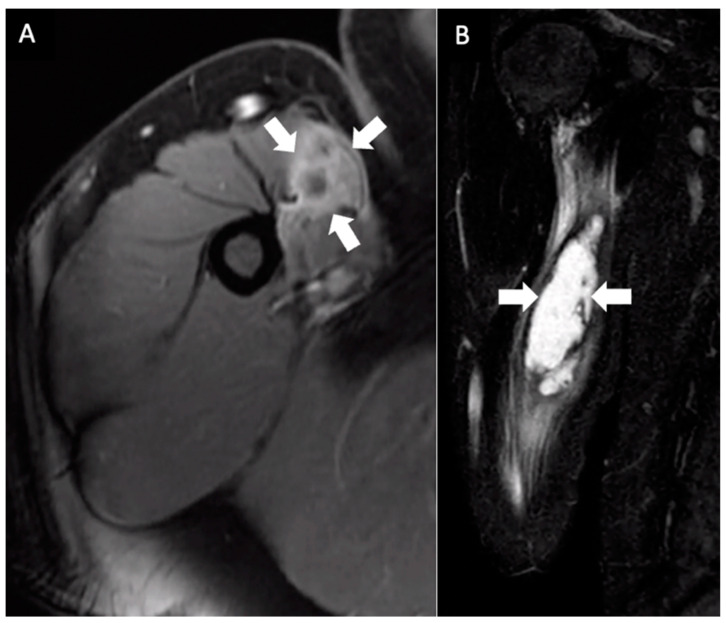
Multiplanar MRI of the right upper arm taken one month after identifying the mass and seven months after receiving the booster dose. Axial fast spin gradient echo postcontrast (**A**) and coronal STIR (**B**) images show an ill-defined central fluid signal measuring 10 × 3.1 × 1.9 cm with prominent peripheral enhancement (arrows).

**Figure 3:**
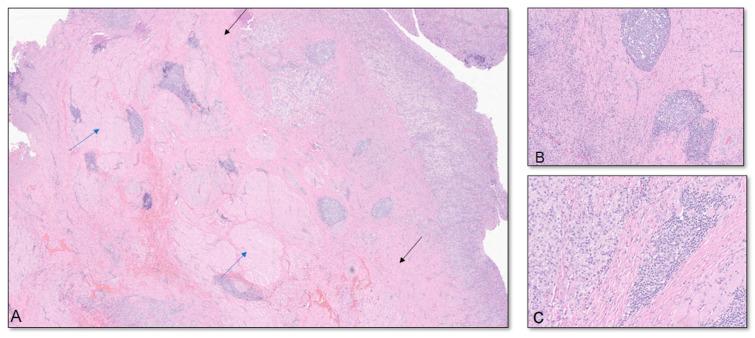
(**A**) Low-power view shows soft tissue and skeletal muscle with mild fibrosis and aggregates of inflammatory cells, consisting mostly of lymphocytes arranged in serpentine and nodular patterns and granulation tissue (blue arrows in the skeletal muscle; black arrows in the fibrous soft tissue). (**B**,**C**) High-power views show the inflammatory infiltrates consisting mostly of lymphocytes, plasma cells, and histiocytes. The cells show no atypia or cytological features of malignancy. Multiple special immunohistochemical studies were performed; Epstein–Barr virus in situ hybridization, Grocott methenamine silver, acid-fast bacillus, and human herpesvirus-8 were negative, ruling out viral and fungal-associated conditions. Lymphoid markers and CyclinD1 were performed and were negative, ruling out hematopoietic or histiocytic neoplasms. Histocytologic findings are most consistent with deep chronic inflammatory tumefaction or inflammatory pseudo-tumor.

His primary care physician referred him to an orthopedic surgeon to evaluate his right upper arm. Physical examination at that time, two weeks later, was notable for a firm, clinically circumscribed, nontender mass in the right upper arm. Right shoulder active range of motion (ROM) was within the normal range, but passive range of motion was decreased. Given the MRI findings of an indeterminate mass, the orthopedic surgeon elected for a needle aspiration. An ultrasound-guided needle aspiration yielded 30 mL of turbid brown fluid, and a biopsy showed fibromuscular tissue with a chronic lymphoplasmacytic inflammatory infiltrate comprised of lymphocytes, plasma cells, and eosinophils. Immediately after the aspiration, there was a subjective improvement in the size of the swelling; as the patient stated, “the mass was not a golf-ball anymore”. However, a second MRI taken two weeks after the needle aspiration showed persistent 10 × 3.1 × 1.9 cm ill-defined fluid signals and peripheral enhancement in the proximal right biceps in the same location as on the first MRI (Figure 2). Given the persistent MRI findings and unclear diagnosis, a complete excision of the mass was accomplished in the following week. The pathology showed soft tissue and skeletal muscle with mild fibrosis and aggregates of inflammatory cells, consisting mostly of lymphocytes, plasma cells, and histiocytes. The cells showed no atypia or cytological features of malignancy (Figure 3).

During follow-up, there was no palpable mass, the pain improved, and his full passive ROM was restored. Six months after excision, a third MRI of the right arm was ordered and showed no residual mass or edema. However, his systemic symptoms of diaphragmatic breathing, fatigue, and reduced exercise intolerance had finally resolved around the time of this final follow-up two weeks following removal of the mass.

This delayed reaction occurred only after receiving the booster dose of the Moderna vaccine. He did not experience a similar reaction to the recommended pediatric and adolescent vaccines or to the initial two doses of the Moderna vaccine.

## 3. Discussion

Cutaneous complications have been mostly associated with mRNA-based vaccines (Pfizer, Moderna) and less frequently with the viral vector vaccines (Ad26.COV2. S (Johnson & Johnson), AZD1222 (AstraZeneca)) [9]. Possible localized adverse events include urticaria and morbilliform rash [9]. Cutaneous reactions from the COVID-19 vaccines can be broken down into type I hypersensitivity (HSR), type IV HSR (DHR), and autoimmune reactions [9]. Type 1 HSR includes urticaria, angioedema, and anaphylaxis [9]. Patients vaccinated with the Pfizer vaccine had the highest frequency of anaphylaxis, followed by the Moderna vaccine [9]. DHR includes facial edema, erythema, dermatitis, and maculopapular and erythema-multiforme-like rashes, which have been strongly associated with the Moderna vaccine but have also been reported with the other types of COVID-19 vaccines [9]. Compared to the Pfizer vaccine, the Moderna vaccine has a greater delay in the onset and resolution of DHR with a longer duration of symptoms [5]. Type IV HSR (DHR) symptomatology is variable, with symptoms usually presenting seven days after the first dose and two days after the second dose and resolving within three days upon receiving any of the COVID-19 vaccines [9]. The third type of cutaneous reactions are autoimmune and adverse events include purpura, lupus erythematous, bullous pemphigoid, and vasculitis [9]. There is very limited data and weak associations between the COVID-19 vaccines and autoimmune reactions [9].

Polyethylene glycol (PEG) is an ingredient of mRNA-based vaccines, which constitutes the major scaffolding to ensure the stability of the vaccines [10]. Reports of cosmetics or drugs containing PEG that had induced cutaneous reactions suggest it as a possible contributor to the COVID-19 vaccine DHR [11]. The detection of anti-PEG immunoglobulins and positive skin pricks with PEG further validated this proposal [11,12]. However, studies have shown an insignificant correlation between anti-PEG immunoglobulins and DHR [13]. Furthermore, the mRNA-based Pfizer vaccine has been shown to be administered safely without inducing DHR [14]. Pfizer and Moderna are both lipid nanoparticle-encapsulated RNAs encoding the severe acute respiratory syndrome coronavirus 2 spike protein [15]. Each vaccine contains a slightly different structural design, which may explain the greater spike-antibody response generated by the Moderna vaccine [15]. Structural differences in the different COVID-19 vaccines are a possible explanation for the greater frequency of side-effects associated with the Moderna vaccine, making the lipid components of the mRNA vaccine another proposed culprit of COVID-19 vaccine DHR [10]. The pathogenicity of lipid nanoparticles is thought to occur due to a complement-mediated activation of the acute phase response [10]. It is difficult to confirm allergic reactions to either of these mRNA vaccine ingredients in our patient as he was never tested for allergy to PEG or lipid nanoparticles. If there is a history of anaphylaxis to any vaccine or allergy to these ingredients, a skin prick test can determine if alternatives to mRNA-based vaccines are indicated [9].

A DHR is a T-cell-mediated reaction that presents with symptoms beginning >4 h after administration [3]. A DHR consists of a pre-sensitized phase and a cytotoxic phase. In the pre-sensitized phase, initial inoculation of a novel antigen is taken up by antigen-presenting cells (APCs), which travel to lymph nodes to present the foreign antigens to immature T-cells. The release of cytokines and chemokines activates T-cells into memory and effector Th1 and Th2 CD4+ cells. After repeat exposure by APCs, the pre-sensitized T-cells release cytokines to activate macrophages and B-cells to stimulate an immune response. In the cytotoxic phase, APCs present foreign antigens to immature T-cells, which stimulate Th1 CD8+ cells. CD8+ cells can create a direct cytotoxic effect through extrinsic mechanisms of apoptosis. If the activated immune cells are unable to phagocytose foreign antigens, macrophages are activated into epithelioid histiocytes to form a granuloma around the pathogens. Epithelioid histiocytes are the hallmark of granuloma formation.

As noted in the pathology reports, the lymphoplasmacytic cells and T-cell and B-cell populations found in the inflammatory infiltrate suggest a chronic state of inflammation with the formation of a granuloma [16]. Although the patient reported that the booster dose was injected into the right deltoid, he presented with a mass in the proximal-anteromedial right biceps. We hypothesize that the APCs containing fragments of foreign antigens traveled through lymphatic vessels of the upper limb and transmigrated into the surrounding tissue prior to draining into the adjacent lymph node, given the absence of lymph node involvement in Figure 3 [17]. It is this secondary edematous response that is hypothesized to have led to the palpable pseudo-tumors in these patients. The other possibility is that the injection missed its mark.

Immediately after receiving the booster dose, the patient developed systemic symptoms of diaphragmatic breathing, fatigue, and reduced exercise tolerance. Despite treatment, his chronic inflammatory state persisted until after the mass was completed excised. These are common symptoms that are seen after vaccination, but our patient had persistence of symptomatology well past the median duration of five days [1]. One study found that the booster dose for all COVID-19 vaccines elicited injection-site pain and lymphadenopathy at a higher rate compared to the first and second doses but a lower rate of fever and fatigue [18].

An orthopedic oncologist’s main concern for a patient presenting with any localized swelling is neoplasm, but the differential diagnosis includes infectious and inflammatory conditions. Delayed inflammatory cutaneous reactions have been reported from vaccination against pertussis, anthrax, and botulinum F toxoid [19,20]. Aluminum salt adjuvants used in the pertussis vaccine produced an inflammatory granulomatous response in up to 77% of children [20]. To date, there have been only two cases reporting inflammatory granulomatous reactions resulting in pseudo-tumors after receiving COVID-19 vaccines [7,8]. The first case discussed a granulomatous soft tissue mass at the injection site following the Moderna booster dose [7]. The soft tissue mass mimicking sarcoma presented similarly on histology but resolved spontaneously [7]. Interestingly, there have been reports of the Moderna vaccine being associated with malignancy. One case described a sarcoma in the right upper arm within days of receiving the second dose of the Moderna vaccine, which may have been initially unrecognized until local symptoms brought attention to it [21], while another case revealed tumor regression after receiving the initial doses of the Moderna vaccine [22]. The variability presented in these cases raises the possibility of immunogenicity and reactogenicity varying based on an individual’s physiology and history of underlying oncologic and orthopedic conditions.

An important aspect of this case is the possible contribution of injection technique, particularly relative to the anatomic location of the mass relative to the recommended injection site. The patient reported that the injection was administered one finger breadth above the deltoid tuberosity. Current vaccination guidelines recommend an appropriately sized needle injected intramuscularly into the bulk of the deltoid muscle, approximately three finger breadths below the mid-acromion [23]. As per guidelines and the patient’s description of his injection, we believe that the needle may have been injected lower in the deltoid than recommended. It is recommended to inject into the middle of the deltoid at its bulkiest area to avoid inoculation into the surrounding bony structures (acromioclavicular joint proximally, deltoid tuberosity distally) or axillary nerve. The upper limb, including the deltoid, contains an extensive network of lymphatic vessels that drain into the adjacent lymph nodes before draining into the central lymph nodes of the axilla. These lymphatic vessels play an important role in the uptake and presentation of inoculated antigens to produce a proper immune response, as is hypothesized to have occurred in this case. Another injection complication is shoulder injury related to vaccine administration (SIRVA), but this is not felt to have occurred in this case [23]. The criterion for SIRVA is the rapid onset of localized pain with limited ROM in the absence of pain prior to injection [24]. The patient’s MRI findings of mild hemorrhaging fluid with decreased passive ROM and 3/10 pain raised concerns of a possible SIRVA complication induced by trauma or injection technique. While SIRVA was in the differential, the surgical pathology report showing histiocytes present in the inflammatory infiltrate allowed us to rule out SIRVA in favor of a granulomatous reaction. Granulomas are not associated with SIRVA but are the hallmark histologic finding of a pseudo-tumorous granulomatous injection. In addition, the patient’s ongoing immune reaction, characterized by persistent fatigue and exertional dyspnea, substantiates the mass as a product of a chronic immune reaction. These systemic symptoms have not been associated with SIRVA [24].

## 4. Conclusions

Pseudo-tumors due to DHR are rare manifestations of COVID-19 vaccination, for which the key vaccine components linked to the granulomatous reactions remain unknown. Clinicians should be aware of the pseudo-tumorous appearance in the context of the history of COVID-19 vaccination in the region of a soft tissue mass. Although uncommon, pseudo-tumor or DHR-associated symptoms are not contraindications to receiving the COVID-19 vaccine.

## Data Availability

All relevant data are provided in the paper.
